# Computational Approaches for Identification of Potential Plant Bioactives as Novel G6PD Inhibitors Using Advanced Tools and Databases

**DOI:** 10.3390/molecules28073018

**Published:** 2023-03-28

**Authors:** Rana M. Aldossari, Aarif Ali, Muneeb U. Rehman, Summya Rashid, Sheikh Bilal Ahmad

**Affiliations:** 1 Department of Pharmacology & Toxicology, College of Pharmacy, Prince Sattam Bin Abdulaziz University, P.O. Box 173, Al-Kharj 11942, Saudi Arabia; 2 Division of Veterinary Biochemistry, Faculty of Veterinary Science and Animal Husbandry, SKUAST-Kashmir, Alustang, Shuhama 190006, Jammu & Kashmir, India; 3 Department of Clinical Pharmacy, College of Pharmacy, King Saud University, P.O. Box 2457, Riyadh 11451, Saudi Arabia

**Keywords:** G6PD, bioactives, docking, ADMET, toxicity, homology, dynamics, CASTp

## Abstract

In glucose metabolism, the pentose phosphate pathway (PPP) is the major metabolic pathway that plays a crucial role in cancer growth and metastasis. Although it has been pointed out that blockade of the PPP is a promising approach against cancer, in the clinical setting, effective anti-PPP agents are still not available. Dysfunction of the G6PD enzyme in this pathway leads to cancer development as this enzyme possesses oncogenic activity. In the present study, an attempt was made to identify bioactive compounds that can be developed as potential G6PD inhibitors. In the present study, 11 natural compounds and a controlled drug were taken. The physicochemical and toxicity properties of the compounds were determined via ADMET and ProTox-II analysis. In the present study, the findings of docking studies revealed that staurosporine was the most effective compound with the highest binding energy of −9.2 kcal/mol when docked against G6PD. Homology modeling revealed that 97.56% of the residues were occupied in the Ramachandran-favored region. The modeled protein gave a quality Z-score of −10.13 by ProSA tool. iMODS server provided significant insights into the mobility, stability and flexibility of the G6PD protein that described the collective functional protein motion. In the present study, the physical and functional interactions between proteins were determined by STRING. CASTp server determined the topological and geometric properties of the G6PD protein. The findings of the present study revealed that staurosporine could be developed as a potential G6PD inhibitor; however, further in vivo and in vitro studies are needed for further validation of these results.

## 1. Introduction

In pentose phosphate pathway (PPP), a key enzyme is the glucose-6-phosphate dehydrogenase (G6PD) that converts glucose-6-phosphate into 6-phosphogluconate in addition to nicotine adenosine dinucleotide phosphate (NADPH) and nucleotide precursors [1]. In all forms of life ranging from prokaryotes to animals, G6PD is a ubiquitous cytosolic enzyme that catalyzes the first rate limiting step of oxidative phase of this pathway [2,3]. NADPH preserves the reduced glutathione by counterbalancing oxidative stress triggered by various oxidant agents [4]. In biosynthetic pathways, NADPH acts as an electron donor for several enzymatic steps and is vital to protect cells from oxidative damage. G6PD monomer has a molecular weight of 59 kDa and consists of 515 amino acids, and the enzyme is active as a dimer or tetramer. Globally, more than 400 million people have G6PD deficiency, which is the most common human enzyme defect reported [5]. A predominant cause of erythrocyte lysis and intravascular hemolysis is the deficiency of G6PD, which leads to NADPH scarcity and henceforth inability to renew glutathione.

Cancer is the most common disease and leading cause of death in the world every year. This disease is multifactorial and shares many features with other cancers, and one of the common hallmarks is reprogrammed energy metabolism [6]. In the development of cancer, protein dysregulation and the PPP flux may be involved. The first rate-limiting step in the PPP pathway also plays a significant part in cancer development, and, in many cancers, there is upregulation of G6PD. Moreover, overexpression of G6PD is also related to stage and degree of cancer, including the tumor size, survival rate, invasion depth, metastasis in lymph node and stage of tumor [7]. The intracellular metabolic pathway is hijacked by cancer cells in order to sustain prompt replication and promote the synthesis of vast important cellular constituents. G6PD overexpression influences DNA synthesis, regulation of cell cycle, DNA repair, metastasis, invasion and redox equilibrium proliferation to provide a beneficial environment to cancer cells [8,9,10,11,12]. The activity and expression of G6PD are strongly regulated through transcription, translation, post-modifications and associations with other proteins [13]. G6PD is highly expressed in different cancers, such as lung adenocarcinoma [14], breast carcinoma [15], colorectal cancer [16], hepatocellularcarcinoma [13,17], glioma [18] and gastric cancer [7]. Similarly, G6PD expression is found to be decreased in acute myeloid leukemia [19], pituitary cancer [20] and melanoma [21].

The efficacy of cancer drugs is affected by G6PD, therefore leading to drug resistance. Computational drug studies have been continuously increased with high success rates as the in silico analysis has become well correlated and more realistic with the actual in vitro studies. In the near future, computational tools will be a fundamental part of the drug discovery studies. Hence, the present study was designed with the aim of finding new bioactive G6PD inhibitors after knowing the dearth of approved clinical medications. Continued efforts to develop therapies and treatments against cancer other than chemotherapy and radiotherapy are being made. However, these treatments further deteriorate health of patients and make them suffer from a lot of stress [22]. Subsequently, natural plant bioactives are more often considered and likely to serve as alternate to existing therapeutic substances used in cancer treatment. For a long time, natural products have been used more commonly to treat and cure human diseases, but, in recent times, the demand to use natural bioactives has greatly increased [23]. The natural constituents obtained from different medicinal plants could be used potentially to treat and protect against cancer. Research studies have reported immune-modulating and anticancer activity of several bioactive compounds with their effects on cancer invasion and metastasis [24].

The recent development in computational field has developed a rationale for identifying potential bioactives that can target G6PD protein. Computational approaches could be potentially used to repurpose natural bioactives for drug designing. Here, in this study, we have used Staurosporine, Withanoside II, Zingerone, Zerumbone, Polydatin, Naringenin, Isosilychristin, Eugenol, Gingerenone and Syringic acid molecules, which could act as potential G6PD inhibitors by using computational methods. In this study, ADMET (absorption, distribution, metabolism, excretion and toxicity) analysis of plant bioactives and the control drug was performed. Further, the present study also aimed to determine docking analysis of the G6PD protein with natural compounds, toxicity analysis in addition to homology modeling, molecular dynamics, protein-protein interactions (PPIs) and topological features of human G6PD protein.

## 2. Materials and Methods

### 2.1. Ligand Selection

In the present study, ligands such as Staurosporine, Withanoside II, Zingerone, Zerumbone, Polydatin, Naringenin, Isosilychristin, Eugenol, Gingerenone and Syringic acid were selected along with the control drug (Lapatinib). An online server, PubChem (https://pubchem.ncbi.nlm.nih.gov/compound; last accessed on 5 December 2022), was used for 2D ligand structure retrieval in a simple data format (SDF).

### 2.2. Online Smiles Translator

The ligands’ smiles retrieved from PubChem server were used to convert ligands from SDF into PDB format by using an online web server (https://cactus.nci.nih.gov/translate/; accessed on 5 December 2022).

### 2.3. ADMET Analysis

To determine ADMET (Adsorption, Distribution, Metabolism, Excretion and Toxicity) of molecules is very important for being used as drug. SWISSADME, an online webserver (www.swissadme.ch/index.php; accessed on 5 December 2022), was used to determine ADMET properties of natural compounds and drug molecules [25,26]. In the SWISSADME server, the smiles of ligands retrieved from PubChem were consequently uploaded. Lipinski’s rule of five parameters are as follows: molecular weight <500 Da, less than 10 hydrogen bond acceptors, hydrogen bond donors not more than 5 and A*log*P should be <5. 

### 2.4. Toxicity Analysis

ProTox-II an online web tool (https://ox-new.charite.de/protox_II/; last accessed on 5 December 2022) was used to evaluate compound toxicity.

### 2.5. Molecular Docking

The three-dimensional structure of G6PD was retrieved from the protein data bank (https://www.rcsb.org/structure/; accessed on 5 December 2022) with the PDB ID of 6JYU. Auto Dock tools (UCSF-Chimera©, version 4.2.6) performed the docking analysis of G6PD protein in this study. The docking method was performed as per the protocol mentioned by [27]. Initially, the protein was prepared in AutoDock tool by removing molecules of water first, followed by addition of polar hydrogen and finally Gasteiger charges. Prior to the preparation of grid, the docked protein molecules were selected as macromolecules. The natural compounds were selected as ligand molecules in the input option and torsion angles were added. Both the protein and ligand molecules were then saved in pdbqt format. In the present study, the grid box size dimensions (x, y, z) were 40 Å each with spacing of 0.375 Å and the center dimensions (x = 9.091, y = 21.618, z = 24.440 Å), respectively.

### 2.6. Inhibition Constant

In the present study, inhibition constant (Ki) was determined from binding energy (ΔG) by using the following formula:

Ki (nM) = exp(ΔG/RT), where T (Temperature = 298.15 K) and R is the gas constant (1.98 × 10^−3^ kcal/mol^−1^ k^−1^).

### 2.7. Structural Analysis

In the structure building of protein, SWISS-MODEL (version 8.9), an easily available online tool (https://swissmodel.expasy.org/; accessed on 5 December 2022), was used [28,29]. This tool requires amino acid sequence of protein in fasta format as an input for modeling building.

### 2.8. ProSAweb

The overall quality and validity of the modeled protein structure was determined by ProSAweb tool (https://prosa.services.came.sbg.ac.at/prosa.php; accessed on 5 December 2022) [30].

### 2.9. PROCHECK

The accuracy of the G6PD protein model was assessed by PROCHECK tool, and the findings were presented in Ramachandran plot (https://saves.mbi.ucla.edu/; accessed on 5 December 2022) [31,32].

### 2.10. iMODS

An easily available online web server that performs normal mode analysis (NMA) on both proteins and nucleic acids (https://imods.chaconlab.org/; accessed on 5 December 2022). This tool computes vibrational modes from various chains of nucleic acids and proteins by using NMA coordinates.

### 2.11. Search Tool for the Retrieval of Interacting Genes/Proteins (STRING)

The protein–protein interactions (PPIs) were predicted by an easily available biological database (STRING) (http://string-db.org/; accessed on 5 December 2022). This tool provides scores wherein predicted confidence scores in edges of each network possess values between 0 and 1, respectively. In the present study, this server was used to assess network summary, gene neighborhood, gene co-occurrence and gene fusion.

### 2.12. Computed Atlas of Surface Topography of Proteins (CASTp)

An online web tool (http://sts.bioe.uic.edu/castp/; accessed on 5 December 2022) measured the geometric and topological features of human G6PD protein. The surface pockets, cross channels and interior cavities in the protein structure are located, delineated and measured by the CASTp server.

## 3. Results

### 3.1. Molecular Docking

**Figure 1:**
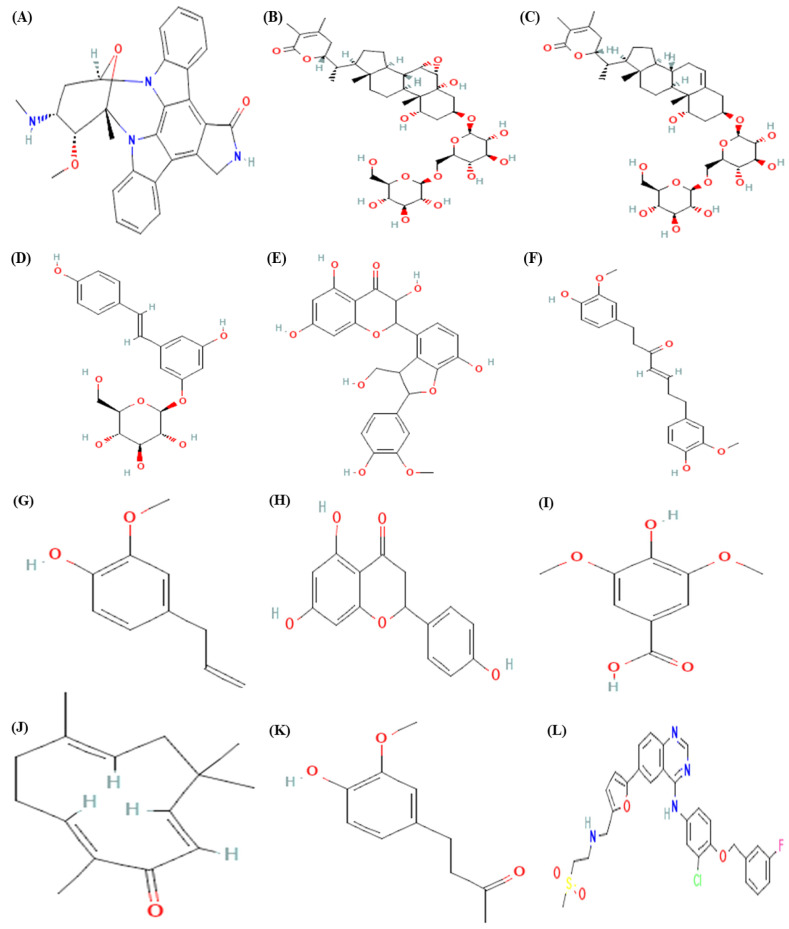
2D structure of bioactive compounds and drug: (**A**) Staurosporine, (**B**) Withanoside II, (**C**) Withanoside V, (**D**) Polydatin, (**E**) Isosilychristin, (**F**) Gingerenone, (**G**) Eugenol, (**H**) Naringenin, (**I**) Syringic acid, (**J**) Zerumbone, (**K**) Zingerone and (**L**) Lapatinib.

The main aim of docking approach is to determine interaction between a protein and a ligand molecule. In this study, ligands such as Staurosporine, Withanoside II, Zingerone, Zerumbone, Polydatin, Naringenin, Isosilychristin, Eugenol, Gingerenone and Syringic acid were selected along with control drug. The 2D structure of bioactive compounds and drug is shown in Figure 1.

In the present study, Staurosporine showed the highest binding affinity of −9.2 kcal mol, followed by Withanoside II (−9.0) and Withanoside V (−8.9), respectively, when docked against human G6PD. Least binding affinity was shown by Zingerone (−5.5 kcal/mol). The results obtained from docking experiments are illustrated in Table 1. The inhibition constant (Ki) µM of all the phytocompounds is shown in Table 1. Staurosporine showed the highest Ki value of 15.54 µM whereas Zingerone reported the lowest (9.29 µM).

**Figure 2:**
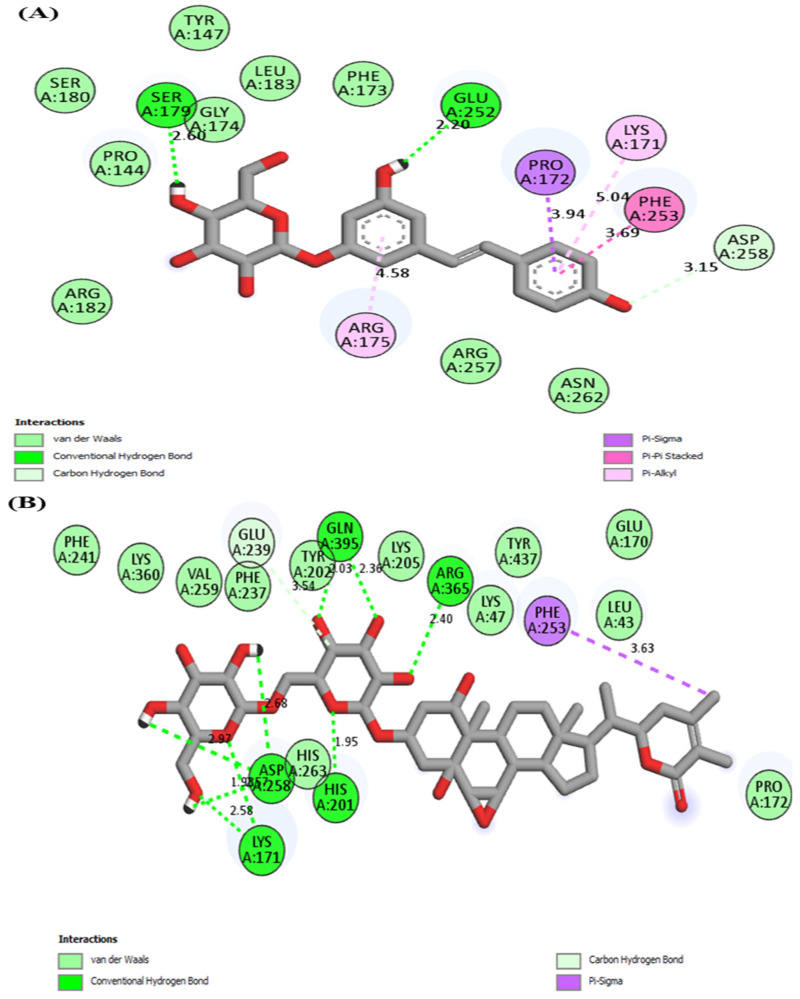

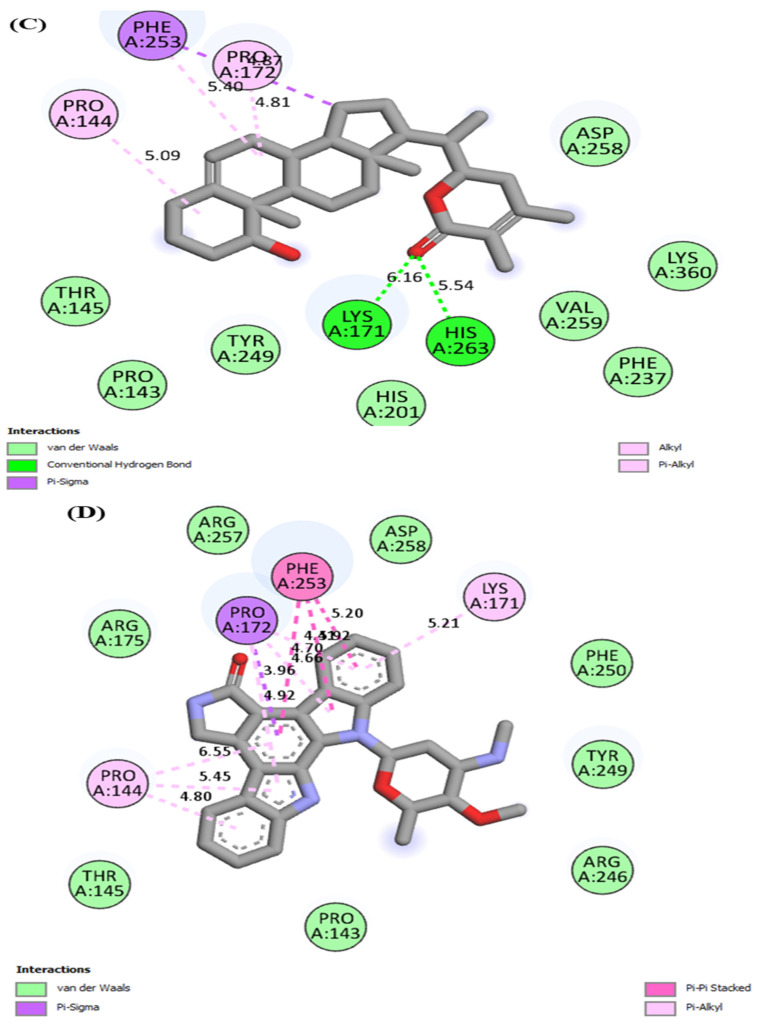

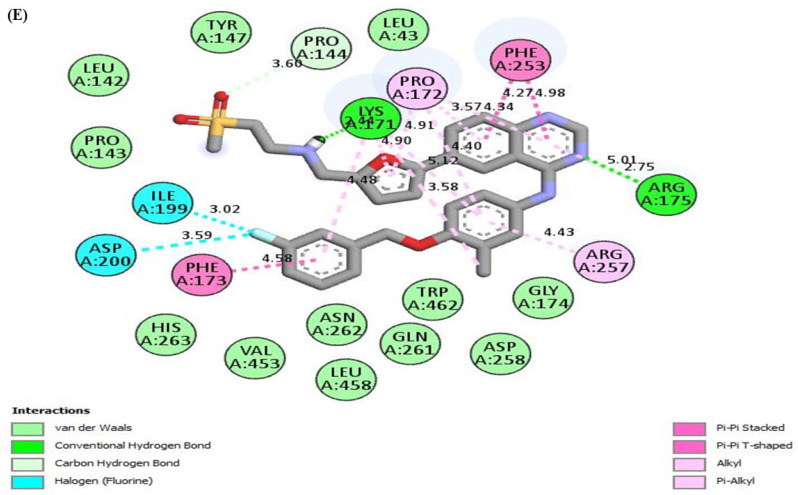
2D Molecular interaction of human G6PD with (**A**) Polydatin, (**B**) Withanoside II, (**C**) Withanoside IV, (**D**) Staurosporine and (**E**) Lapatinib.

The 2D interaction of G6PD protein with four best docked compounds and a standard drug (Lapatinib) is graphically shown in Figure 2.

In the present study, Table 2 illustrates the interacting residues and their positions between docked protein and ligand molecules.

### 3.2. ADMET Analysis

In the present study, the top four compounds that showed best results for docking were further analyzed for ADMET studies. In the present study, physiochemical and drug-like properties of phytocompounds were evaluated based on Lipinski’s rule of five parameters. The phytocompounds that yielded good LOGP/lead likeness/bioavailability, synthetic accessibility, high gastrointestinal absorption, blood–brain barrier (BBB) permeability were selected. In the present study, withanoside II and withanoside IV did not follow Lipinski’s rule and showed three violations, i.e., molecular weight greater than 500 g/mol, number of hydrogen bond acceptors (>10) and donors (>5). In this study, Polydatin and Staurosporine followed Lipinski’s rule and withanoside II and withanoside IV did not. The ADMET results are shown in Table 3.

### 3.3. ProTox-II

**Figure 3:**
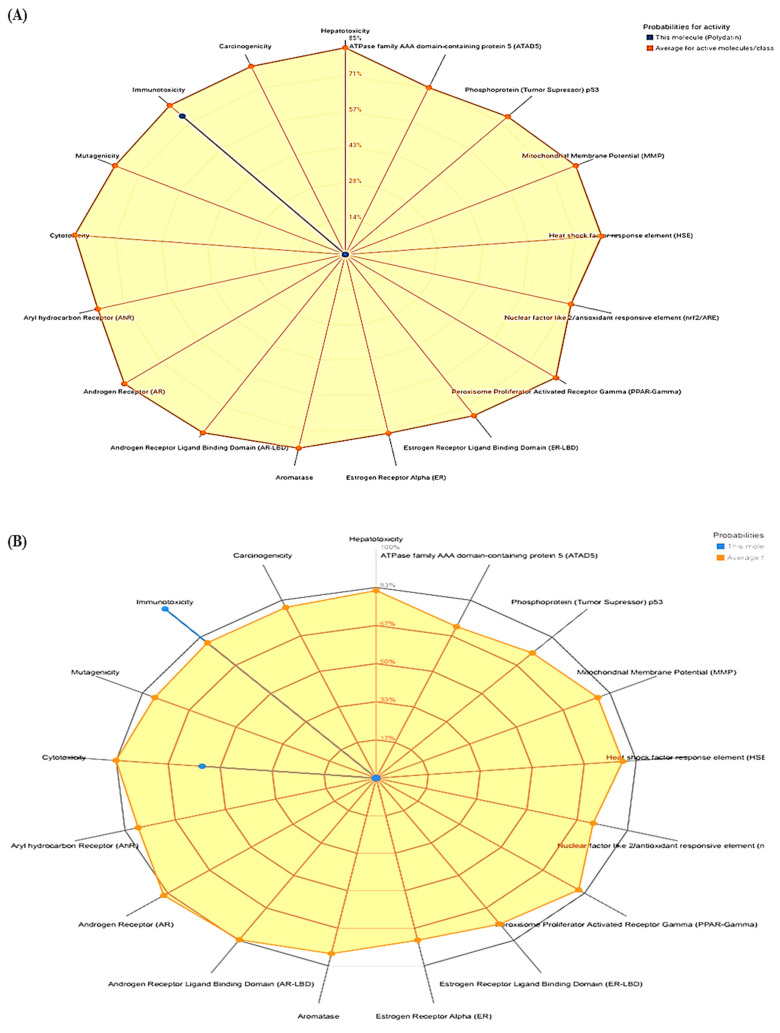

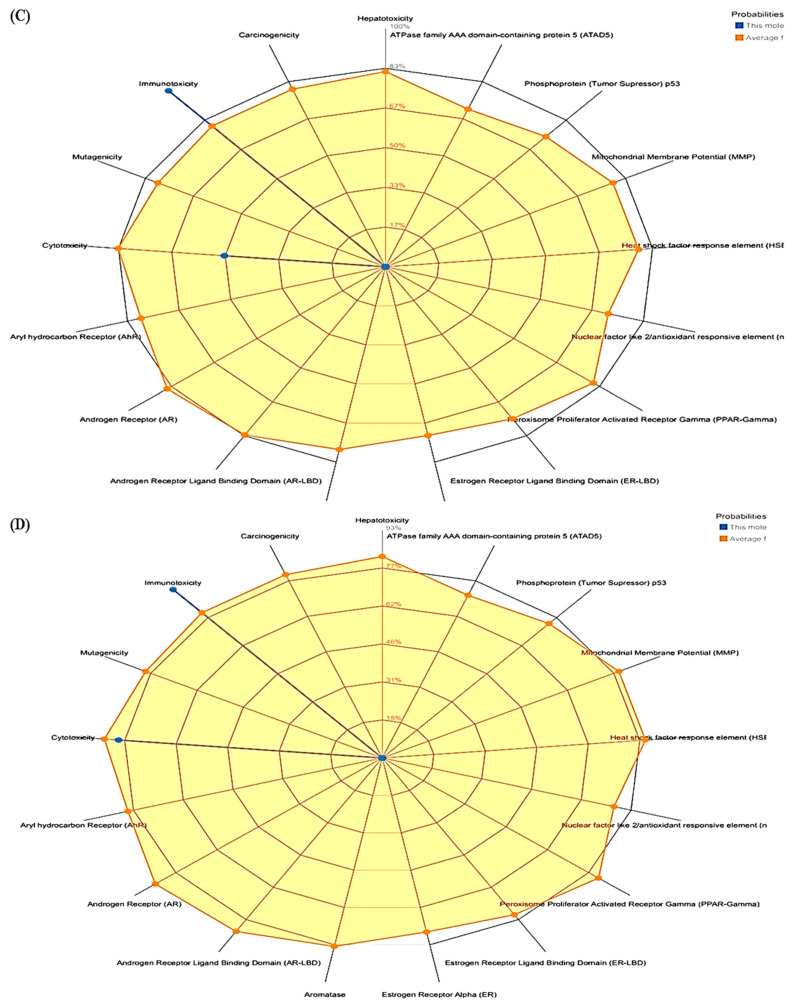

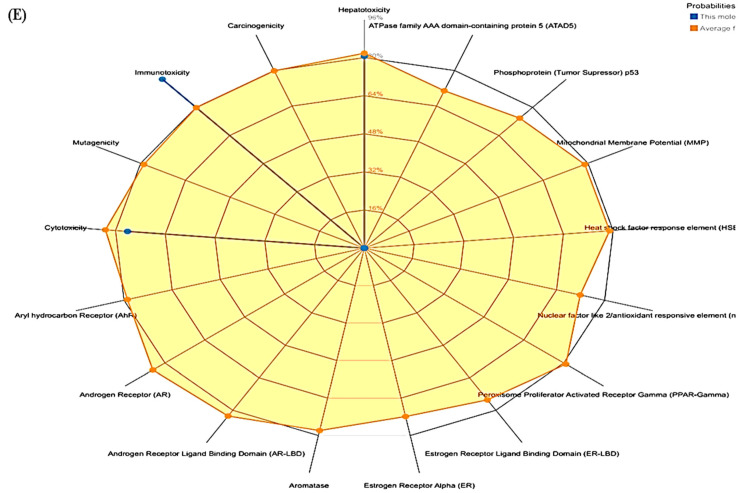
Radar chart of toxicities of phytocompounds and drug by ProTox-II (**A**–**E**).

Different factors were considered for studying toxicity, such as lethal dose 50 (LD_50_), prediction of toxicity class, hepatotoxicity, cytotoxicity, immunotoxicity, mutagenicity and carcinogenicity. Levels of toxicity were categorized as classes 1 and 2 (fatal when swallowed), class 3 (when swallowed could be toxic), class 4 (harmful if swallowed), class 5 (may be harmful if swallowed) and class 6 (non-toxic). The toxicity of the target compounds predicted is mainly based on 15 different targets from the Novartis in vitro safety panels of the target proteins associated to adverse drug reactions. The calculated toxicity targets information and the results are displayed as toxicity radar plots compares the average confidence score of the active compounds in given model training group to that of the taken input compound. The prediction of hepatotoxicity is estimated via data collected from drug induced liver injury and national institute of health liver toxicity database. Similarly data for prediction of carcinogenicity is obtained from carcinogenic potency database (CPDB). Moreover the data is collected from Ames test for prediction of mutagenicity and Chemical European Biology Laboratory (ChEMBL) for cytotoxicity. Further for immunotoxicity prediction, information is retrieved from U.S National Cancer Institute’s (NCI) database. Based on respective LD_50_ (mg/kg), active toxicity was shown by compounds for several toxicities, i.e., Polydatin (immunotoxicity), Withanoside II, Withanoside IV and Staurosporine (cytotoxicity, immunotoxicity). Moreover, the standard drug Lapatinib showed active toxicity for hepatotoxicity, cytotoxicity and immunotoxicity. The organ toxicity of Polydatin is predicted with a confidence score of 0.85 and is predicted to be active for hepatotoxicity. Similarly, the organ toxicity for Withanoside II, Withanoside IV and Staurosporine was predicted to have a confidence score of 0.55, 0.50 and 0.79 and predicted to be active for cytotoxicity and confidence score of 0.99 and 0.92 and predicted to be active for immunotoxicity. Moreover, for the drug Lapatinib, the predicted confidence score of 0.85, and 0.74 was found and hence reported to have active toxicity for hepatotoxicity, cytotoxicity and immunotoxicity. The results of toxicity obtained from ProTox-II are shown in Table 4 and Figure 3.

### 3.4. Structural Analysis

**Figure 4:**
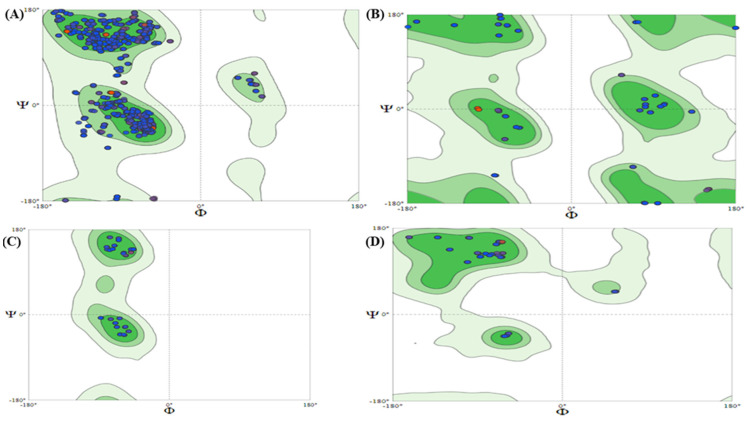
Ramachandran Plots of G6PD: (**A**) All chains, (**B**) Glycine, (**C**) Proline and (**D**) Pre-proline.

In this study, human G6PD was shown. In the background, the ϕ and ψ values of the residues are plotted (Figure 4). In the Ramachandran-favored region lay 97.56% of the residues. The clash score for residues was 0.66, for Ramachandran outliers (0.21%) and 2.25% for rotamer outliers. Similarly, in the human G6PD protein, there were 16 C-beta deviations, 77 bad angles and 4 bad bonds, respectively (Table 5). The favored regions are shown in green color, generously allowed regions in pale green, additional allowed regions in light green and disallowed regions in white. The values for Gly, Pro and pre-Pro residues are shown in Figure 4. 

**Figure 5:**
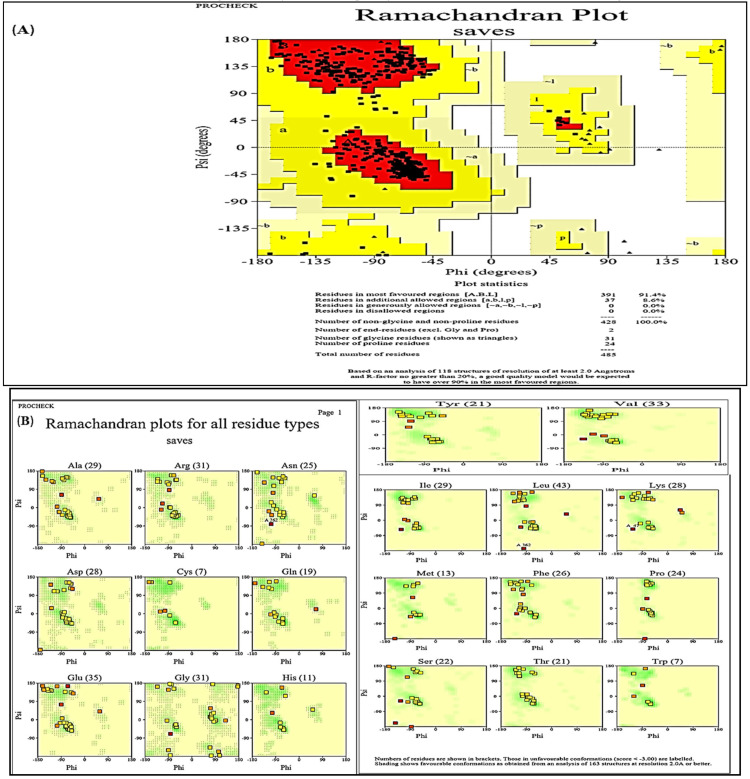
PROCHECK analysis of Ramachandran plot with (**A**) Plot statistics and (**B**) All residues.

PROCHECK web server was used to evaluate and determine the three-dimensional structure of G6PD protein, which assessed Ramachandran plot and provided results for residues presenting regions with different colors, i.e., favored (red), additionally allowed (yellow), generously allowed (pale yellow) and disallowed (white) (Figure 5). 

The overall quality and structure validity of p53 protein were determined by a ProSA tool, which showed overall model quality Z-score of −10.13. The Z-score predicts the complete energy deviation of a protein structure as compared to the distributed energy attained randomly.

**Figure 6:**
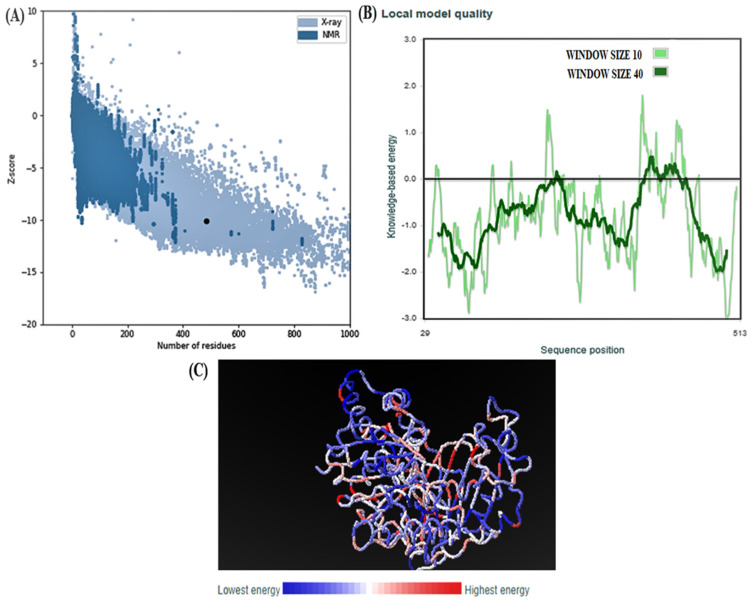
Human G6PD domain Z-score and plot of residue scores. (**A**) ProSA-web *z*-scores of all protein chains. (**B**) Energy plot. (**C**) Highest and Lowest energies of protein.

The Z-score measures the protein structures total energy deviation in comparison to energy distribution acquired from random conformations. The findings obtained from ProSA server are illustrated in Figure 6. The group structures’ results retrieved from different sources (X-ray, NMR) are represented by different colors (Figure 6A). The chains are illustrated by the plots which have a residue size below 1000. Residue energies averaged over a sliding window are plotted as a function of the central residue in the window. A window size of 10 is used due to the large size of the protein chain (default: 40) (Figure 6B). Residues are colored from blue to red in the order of increasing residue energy (Figure 6C).

### 3.5. iMODS

**Figure 7:**
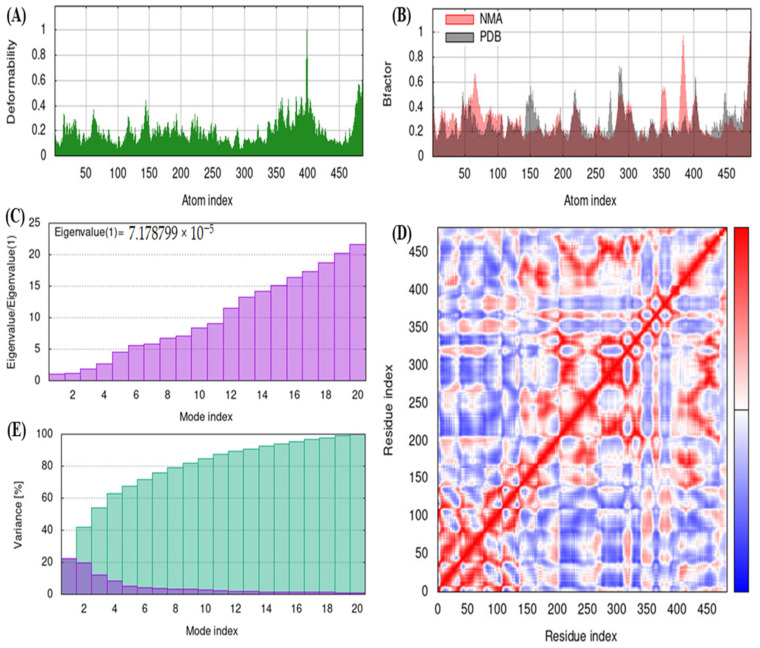
Molecular dynamics. (**A**) Deformity analysis, (**B**) B-factor, (**C**) Eigen values, (**D**) Covariance map and (**E**) Variance.

In this study, iMODS server was used to study deformity, B-factor, eigenvalues, correlation map and variance, and the results obtained are illustrated in Figure 7.

The peaks in the graph represent the areas with deformability (main-chain deformity) in the protein (Figure 7A). In the protein, the hinges were not critical and hence the structure remained stable and the areas with high deformability depict the chain hinge’s location. In this study, B-factor determines the capacity of the molecule to deform at its every residue (Figure 7B). The B-factor analysis showed no substantial fluctuations, thus indicating fewer loops. The motion stiffness of the molecule is represented by the eigenvalues that are associated with every normal mode (Figure 7C). In the present study, the eigenvalue found was 7.178799 × 10^−5^ (Figure 7C). Higher eigenvalues are associated with higher variance whereas lower eigenvalues indicate simple deformation. Figure 7D represents the covariance map estimated by Cartesian coordinates Cα, wherein red color indicates correlation motion of residues, blue (anti-correlated) and white (uncorrelated), respectively. In the variance plots, the colored bars indicate variances, with individual variances represented by red color and cumulative variances by green color (Figure 7E).

### 3.6. STRING

**Figure 8:**
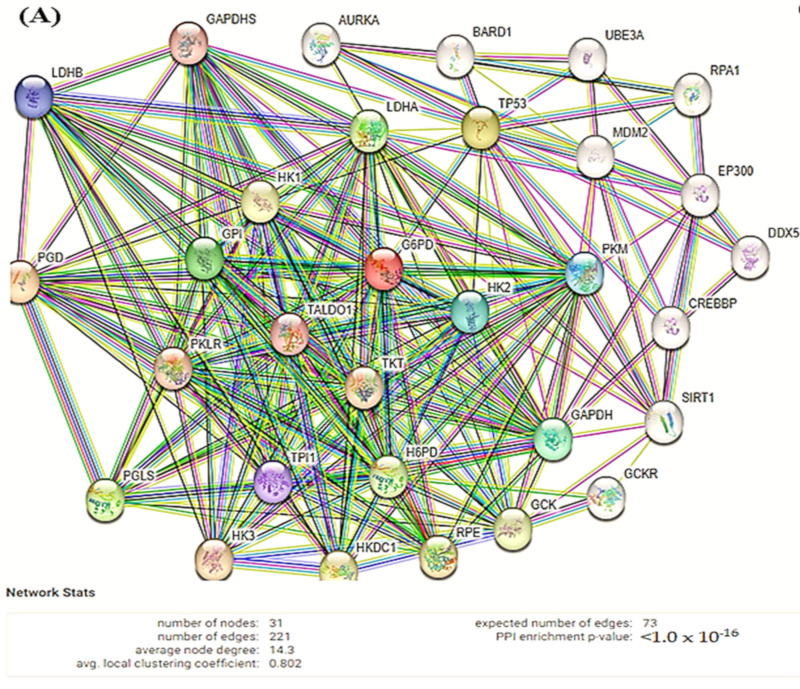

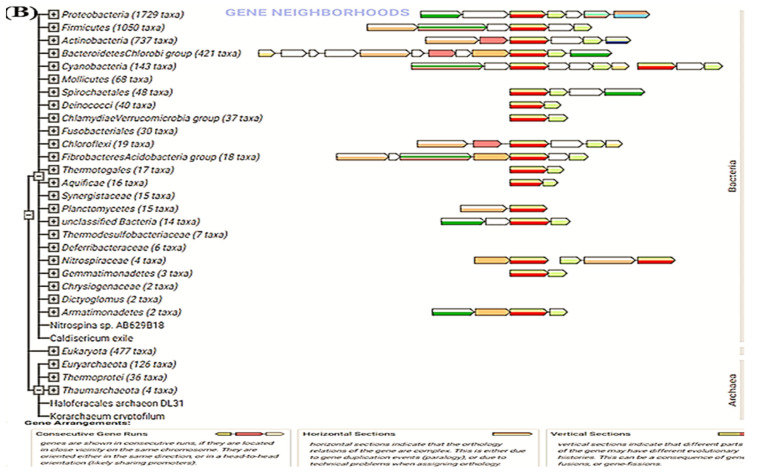

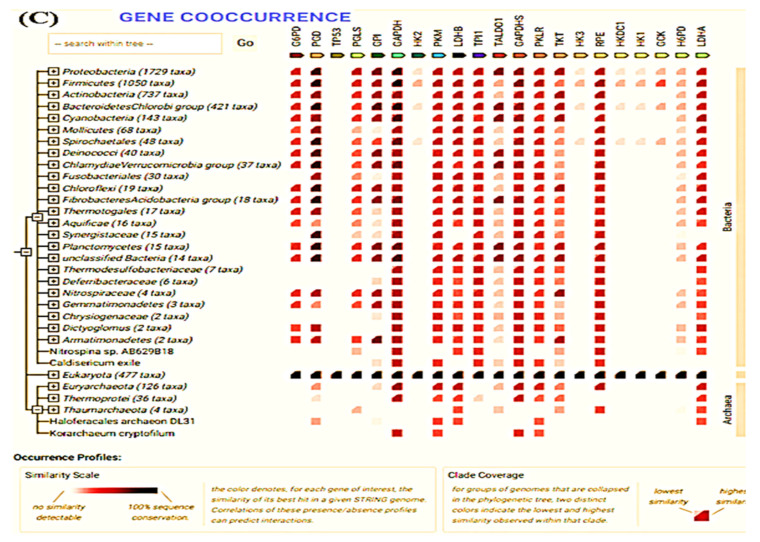

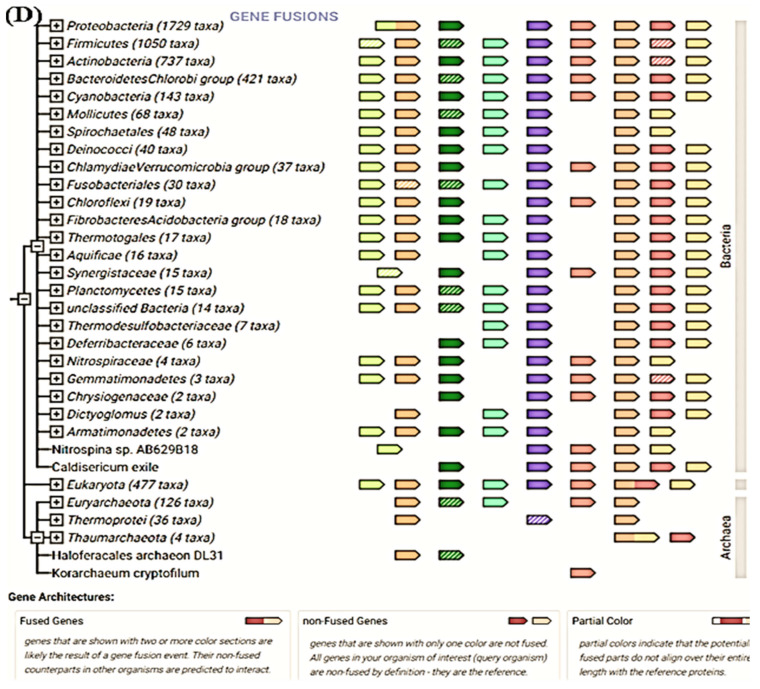
STRING analysis. (**A**) Network interactions, (**B**) Gene-neighborhood, (**C**) Gene Co-occurrence and (**D**) Gene fusion.

In this study, STRING database was used to predict associations between proteins, including functional as well as physical interactions. In the present study, Figure 8A illustrates a typical network association of G6PD protein, wherein 10 more additional proteins were added to expand the network. The line colors indicate specific lines of evidence connecting the protein nodes that are involved in forming functional association. For a particular protein group, the network of associated predicted associations is summarized by the network view (Figure 8A). The line thickness in the confidence mode represents the confidence prediction of the interaction. Figure 8B illustrates the gene neighborhood describing the runs of genes that repeatedly occur in close neighborhood. The black line links the genes located together in a run, whereas white spaces separate the multiple runs for a given species, and other genes below the current threshold are drawn as small white triangles (Figure 8B). Moreover, the gene co-occurrence represents the absence or presence of associated proteins across the species. In different organisms, the intensity of the color indicates the extent of protein similarity, as shown in Figure 8C. In the present study, Figure 8D illustrates the individual events of gene fusion per species. The species listed to the left are the ones in which fusion has occurred.

**Figure 9:**
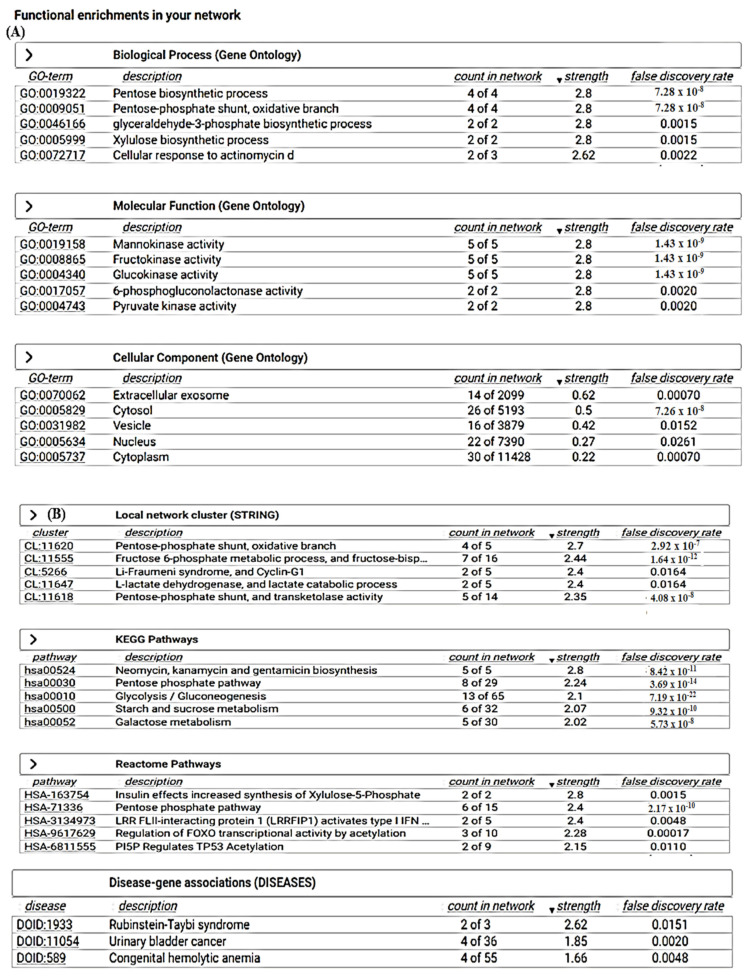
Functional enrichment of human G6PD protein network.

The findings of functional enrichments of human G6PD protein as generated by string server are illustrated in Figure 9 that represents and groups statistical enrichment observations for a number of pathways and functional systems. The functional enrichment analysis determines that whether some functions in a set of differentially expressed genes are enriched. The count in the network indicates the number of proteins in the network that are annotated with a specific term. The second number indicates how many proteins have this term assigned to them in total (in network and in the background). The strength (*Log*10 (observed/expected) describes the magnitude of the enrichment effect. It is the ratio of the number of proteins in the network that are annotated with a term to the number of proteins in a random network of the same size that is expected to be annotated with this term. The false discovery rate describes the significance of the enrichment and Benjamini-Hochberg procedure is used to correct p-values for multiple testing within each category.

### 3.7. CASTp 

**Figure 10:**
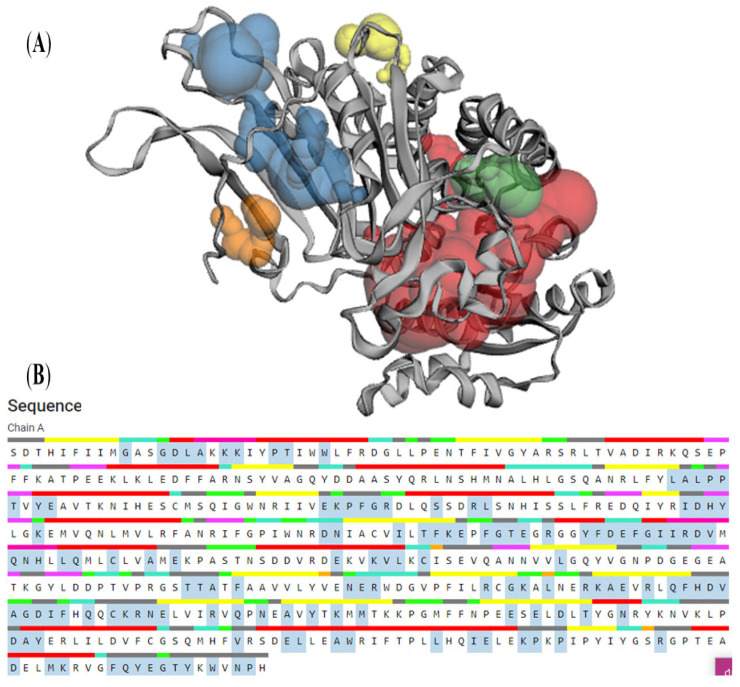
CASTp analysis. (**A**) Pocket panel and (**B**) Sequence panel.

In the present study, the exact area, volume and well as mouth size were determined. Figure 10 shows the top five imprints of the pockets as shown in red, blue, green, orange and yellow, and the atoms forming the pockets as illustrated as sticks. Table 6 shows the volume, area and the pocket ID associated with G6PD protein.

## 4. Discussion

Clinically, in the world, the most prevalent X-linked enzymopathy is the G6PD deficiency. G6PD is a typical housekeeping enzyme, and its severe deficiency in animal models has been found intolerant for growth and development [33,34,35,36] whereas a slight increase in enzyme activity supports a healthy lifespan [37]. In various types of cancers, such as breast, gastric, bladder, ovarian, prostrate, colorectal, leukemia, esophageal and lung, the activity of G6PD is increased [38,39,40,41,42,43,44,45,46,47]. G6PD participates in redox signaling, it affects survival, and death of cells, particularly in diseases such as cancer, and thus G6PD can be exploited as a potential drug target. The currently available medications cause adverse side effects and also affect patient health status. In such a scenario, natural bioactives isolated from plants can be repurposed by computational approaches and henceforth can be used as potential molecules in drug discovery and development.

In the development of new generation inhibitors, molecular docking plays an important role. Prominent insights about protein structure and dynamics are provided by computational approaches in addition to identifying novel therapeutic agents. In the present study, molecular docking analysis revealed that Staurosporine was the most effective compound that showed highest binding affinity of −9.2 kcal/mol against human G6PD protein. This can be explained by the fact that Staurosporine is a competitive inhibitor of protein kinases that binds in the binding pockets of target kinases by competing with ATP molecule [48]. Similar findings have been reported by various studies regarding the ability of Staurosporine as an effective antitumor molecule with potential activity seen in various cancer cell lines (breast, colon, cervical and oral) [49,50,51]. In vitro analysis has revealed that Staurosporine induces apoptosis in many cell lines [52]. In the apoptosis of cancer cells, various signaling pathways have been involved, including mitogen-activated protein kinase (MAPK), cyclooxygenase-2 (COX-2), Janus kinase (JAK), phosphatidylinositol 3-kinase (PI3K), signal transducer and activator of transcription (STAT3) and endoplasmic reticulum stress [53,54,55,56,57,58,59].

In the present study, natural bioactives such as Polydatin, Withanoside II, Withanoside IV and Staurosporine were selected. In the process of drug discovery, a great interest lies in the identification, synthesis and purification of compounds. The physiochemical properties of phytocompounds were determined by ADMET analysis. The evaluation of drug-like properties are particularly important as they are associated with dissolution and intestinal permeability [60,61,62]. Bioactive compounds cannot be used as drug moieties if they fail in more than two rules, as mentioned by Lipinski [60]. During research trials, drug breakdown is often caused by the toxicity of drugs, which is primarily due to unfavorable effects [63].

In the drug development process, it is particularly very important to determine the compound toxicities, and, in such a scenario, ProTox-II is an important computational tool [64]. The prediction of compound toxicities is analyzed much faster by computational methods in comparison to animal experimentation as these in silico approaches provide results in a minimal time span [65].

In the present study, homology modeling was used to estimate 3D structure of human G6PD from its amino acid sequence. In the drug discovery process, homology modeling has been applied successfully as drugs interact with the receptor (protein) as this approach has made a significant contribution in lowering the gap between the known sequences of protein and the experimental structures. The Ramachandran plot is important as it predicts the correctness and quality of the protein structures. The observations of the present study depict the protein model to be stable and stereochemically possible. In homology and comparative studies, modeling methods have proven to be a potential acquaintance to the structural studies when the experimental results are unavailable [66]. In SWISS-MODEL, the complete structure and stoichiometry of protein model depends upon biologically related surfaces that are probably likely to vary less than the remaining surface of protein [67,68]. The QMEAN-Z scores help to generate quality estimates on a local and global scale (Benkert et al., 2011). Therefore, the quality of the protein model can be evaluated by determining model variability by considering the exact properties of the protein structure that are more conserved [69,70].

In the present study, the iMODS tool determined the molecular dynamics of the protein molecule using NMA procedure. The deformable areas in the protein model were not vital, and hence the structure remained quite stable. B-factors indicate the flexible areas/binding sites and are related to atom packing in the protein molecule [71,72,73]. The motion of the protein molecule is represented by the eigenvalues that are directly proportional to the amount of energy needed to deform protein structure. The lower eigenvalues indicate easier deformation and vice versa. The co-variance map represents the several pairs of anti-correlated and correlated residues as represented by white, red and blue colors that depict coupling of residue pairs. STRING helps to reveal protein–protein interactions (PPIs) that subsequently determine the function of target protein molecule. In the identification of new therapeutic drug molecules, PPIs provide vital insights [74].

In the present study, CASTp revealed the topological and geometric properties that are particularly important for protein structures to carry out their functions, such as DNA interaction, ligand binding and enzymatic activity. The determination of topological properties is significant in understanding the structure–function association of proteins, developing therapeutics against targets and manufacturing proteins with desired characteristics [75,76,77].

## 5. Conclusions

G6PD is the most prominent enzyme-linked deficiency whose activity is elevated in different types of cancer. Due to adverse effects caused by medications, natural bioactives have shown immense potential because of their diverse composition. Molecular docking was used to determine the ability of plant bioactives to reveal their inhibitory activity against human G6PD. Our study revealed that Staurosporine was the most effective molecule against G6PD. The physiochemical properties of the natural compounds were determined by ADMET studies. The toxicity of the compounds was estimated by ProTox-II, with many compounds showing active activity for various classes of toxicities. Molecular modeling revealed the quality of protein model, with 97.56% of the residues occupying the Ramachandran-favored region. MolProbity score predicted the quality and correctness of the modeled G6PD protein structure. In cellular systems, protein–protein interactions play a significant role, and the string server determined the physical and functional associations. The molecular dynamic approach was used to reveal the behavior of G6PD protein. CASTp determined the topological properties in the G6PD protein structure as significant for the protein molecule to perform its function. The present study demonstrated that Staurosporine could be an effective bioactive molecule to tackle G6PD-related diseases. However, in vivo and in vitro studies are further required to validate the role of this compound to diseased states. Thus, based on the observations, we can conclude that Staurosporine can prove a potent and efficacious compound that interacts with G6PD protein and can be further repurposed as a drug candidate.

## Figures and Tables

**Table 1:** Binding affinity (kcal/mol) of natural compounds.

S. No	Phytocompounds	Binding Affinity (kcal/mol)	Inhibition Constant (Ki) µM
1	Staurosporine	−9.2	15.54
2	Withanoside II	−9.0	15.20
3	Withanoside V	−8.9	15.03
4	Polydatin	−8.5	14.36
5	Isosilychristin	−8.2	13.85
6	Gingerenone	−7.8	13.17
7	Eugenol	−7.6	12.84
8	Naringenin	−7.2	11.86
9	Syringic Acid	−7.2	11.86
10	Zerumbone	−6.2	10.47
11	Zingerone	−5.5	9.29
Drug			
1	Lapatinib	−7.9	13.34

**Table 2:** Interaction and position of interacting residues between protein and compounds.

Protein	Compound	Interaction	Position
G6PD	Polydatin	Hydrogen bonding	SER179, GLU252
		Van der waals	PRO144, TYR147, PHE173, GLY174, SER180, ARG182, LEU183, ARG257, ASN262
		Pi-alkyl/alkyl	LYS171, ARG175
		Pi-sigma	PRO172
		Pi-Pi stacked	PHE253
		Carbon hydrogen bond	ASP258
	Withanoside II	Hydrogen bonding	LYS171, HIS201, ASP258, ARG365, GLN395
		Van der waals	LEU43, LYS47, GLU170, TYR437, PRO172, HIS263, LYS205, TYR202, PHE237, VAL259, LYS360, PHE241
		Pi-sigma	PHE253
		Carbon hydrogen bond	GLU239
	Withanoside IV	Hydrogen bonding	LYS171, HIS263
		Van der waals	PRO143, THR145, HIS201, TYR249, VAL259, PHE237, LYS360, ASP258
		Pi-alkyl	PRO144, PRO172
		Pi-sigma	PHE253
	Staurosporine	Van der waals	THR145, PRO143, ARG246, TYR249, PHE250, ARG175, ARG257, ASP258
		Pi-alkyl	PRO144, LYS171
		Pi-sigma	PRO172
		Pi-Pi stacked	PHE253
	**Drug**		
	Lapatinib	Hydrogen bonding	LEU142
		Van der waals	ASP42, LEU140, ALA141, TYR147, ARG175, TRP462, PHE173, ASP258, PHE253, TYR249, ARG246
		Pi-alkyl	PRO172
		Pi-anion	GLU170
		Amide-pi stacked	LYS171
		Pi-sigma	LEU43
		Halogen	GLY174
		Carbon hydrogen bond	ARG257

**Table 3:** ADMET properties of phytocompounds.

ADMET	Compounds				Drug
	Polydatin	Withanoside II	Withanoside IV	Staurosporine	Lapatinib
	Physicochemical Properties				
Molecular weight (g/mol)	390.38	798.91	782.91	466.53	581.06
Topological polar surface area (TPSA) (Å^2^)	139.84	257.82	245.29	69.45	114.73
Num. of H bond acceptors	8	16	15	4	8
Num. of H bond donors	6	9	9	2	2
Molar Refractivity	100	193.69	194.21	139.39	153.88
XLOGP	1.03	0.12	0.99	3.24	5.12
iLOGP	1.75	4.80	3.58	3.29	4.20
MLOGP	−0.36	−1.67	−1.03	2.60	3.44
WLOGP	0.23	−0.98	−0.19	3.39	7.34
Lipinski	Yes	No	No	Yes	Yes
Veber	Yes	No	No	Yes	No
Ghose	Yes	No	No	No	No
Egan	No	No	No	Yes	No
Muegge	No	No	No	No	No
Bioavailability score	0.55	0.17	0.17	0.55	0.55
GI absorption	High	Low	Low	High	Low
BBB permeability	No	No	No	Yes	No
P-gp substrate	Yes	Yes	Yes	Yes	No
CYP1A2 inhibitor	No	No	No	No	No
CYP2C19 inhibitor	No	No	No	Yes	Yes
CYP2C9 inhibitor	No	No	No	No	Yes
CYP2D6 inhibitor	No	No	No	Yes	Yes
CYP3A4 inhibitor	No	No	No	Yes	Yes
Log Kp (skin permeation) cm/s	−7.95	−11.09	−10.37	−6.85	−6.21
Pan-assay interference compounds (PAINS)	0	0	0	0	0
BRENK	1	2	1	0	0
Leadlikeness	No	No	No	No	No
Synthetic accessibility	4.82	8.89	8.88	4.93	4.05

**Table 4:** Toxicity analysis of natural compounds and drug.

Compounds	LD_50_ (Mg/Kg)	Toxicity Class	Prediction Probability				
			Hepatotoxicity	Cytotoxicity	Immunotoxicity	Mutagenicity	Carcinogenicity
Polydatin	1380	4	0.85	0.85	0.74	0.73	0.81
Withanoside II	3	1	0.93	0.55	0.99	0.80	0.72
Withanoside IV	19	2	0.94	0.50	0.99	0.96	0.74
Staurosporine	1000	4	0.73	0.79	0.92	0.52	0.61
Lapatinib	1500	4	0.80	0.76	0.96	0.51	0.55

**Table 5:** Stereochemical properties and interacting residues of human G6PD protein.

S. No	Parameters	Interacting Residues
1	Ramachandran outliers	D407 ASP, B407 ASP, A407 ASP, C407 ASP
2	Rotamer outliers	D105 GLN, C317 GLU, A317 GLU, B317 GLU, D270 VAL, C270 VAL, B270 VAL, A270 VAL, C150 GLN, B150 GLN, B174 TYR, A174 TYR, D174 TYR, C174 TYR, B395 THR, A395 THR, D395 THR, C395 THR, C393 ASP, D393 ASP, D476 GLU, C476 GLU, B393 ASP, B476 GLU, A393 ASP, A476 GLU, D347 ASP, B347 ASP, A347 ASP, C347 ASP, D407 ASP, C407 ASP, A407 ASP, B407 ASP, A71 GLU, B71 GLU, C71 GLU, D71 GLU
3	C-beta deviations	A216 GLU, B216 GLU, C216 GLU, D216 GLU, B407 ASP, C407 ASP, A407 ASP, D407 ASP, D280 TYR, B280 TYR, C280 TYR, A280 TYR, A151 SER, C151 SER, B151 SER, D151 SER
4	Bad Angles	B479 TYR, A479 TYR, D479 TYR, C479 TYR
5	Bad Bonds	(D438 THR-D439 PRO), (A438 THR-A439 PRO), (B438 THR-B439 PRO), (C438 THR-C439 PRO), (B21 TYR-B22 PRO), D190 ASN, B190 ASN, C190 ASN, A190 ASN, (D21 TYR-D22 PRO), C2 ASP, (A21 TYR-A22 PRO), (C21 TYR-C22 PRO), B2 ASP, A2 ASP, D225 PHE, D2 ASP, C225 PHE, A225 PHE, B225 PHE, (A57 GLU-A58 PRO), D424 PHE, (D57 GLU-D58 PRO), (C57 GLU-C58 PRO), (B57 GLU-B58 PRO), A424 PHE, C424 PHE, B424 PHE, B346 HIS, (A143 LYS-A144 PRO), A346 HIS, D346 HIS, (B143 LYS-B144 PRO), (C143 LYS-C144 PRO), D101 HIS, C346 HIS, B173 HIS, (D143 LYS-D144 PRO), A101 HIS, D235 HIS, B127 HIS, C235 HIS, C173 HIS, B101 HIS, A173 HIS, D173 HIS, C127 HIS, C101 HIS, B235 HIS, D127 HIS, A127 HIS, A235 HIS, C148 ASP, C51 ASP, C354 HIS, D354 HIS, (D448 LYS-D449 PRO), B148 ASP, (B448 LYS-B449 PRO), D158 HIS, B51 ASP, A158 HIS, D51 ASP, C158 HIS, A354 HIS, B158 HIS, C442 HIS, B354 HIS, A51 ASP, (D300 VAL-D301 PRO), B442 HIS, (C300 VAL-C301 PRO), (B300 VAL-B301 PRO), (A300 VAL-A301 PRO), (C369 ASN-C370 GLU), A423 HIS, (D369 ASN-D370 GLU)

**Table 6:** Topological feature analysis by CASTp.

Pocket ID	Area	Volume
1	1124.12	1501.13
2	508.06	279.43
3	170.81	86.03
4	103.32	33.28
5	55.87	20.12

## Data Availability

All the data generated has been published in this manuscript.

## Abbreviations

PPP: Pentose phosphate pathway; G6PD: Glucose-6-phosphate dehydrogenase; NADPH: Nicotine adenosine dinucleotide phosphate; ADMET: Absorption, Distribution, Metabolism, Excretion and Toxicity; PPIs: Protein-Protein interactions; SDF: Simple data format; NMA: Normal mode analysis; CASTp: Computed atlas of surface topography of proteins; BBB: Blood-brain barrier; TPSA: Topological polar surface area; GI: Gastrointestinal absorption; PAINS; Pan-assay interference compounds; LD_50_; Lethal dose 50; MAPK: Mitogen activated protein kinase; COX-2: Cyclooxygenase-2; JAK: Janus kinase; PI3K: phosphatidylinositol 3-kinase; STAT3: Signal transducer and activator of transcription; 3D: Three dimensional.
